# Persistence of pregabalin treatment in Taiwan: a nation-wide population-based study

**DOI:** 10.1186/s10194-020-01123-4

**Published:** 2020-05-19

**Authors:** Yen-Feng Wang, Yung-Tai Chen, Ching-Wen Tsai, Yu-Chun Yen, Yi-Chun Chen, Ben-Chang Shia, Shuu-Jiun Wang

**Affiliations:** 1 Department of Neurology, Neurological Institute, Taipei Veterans General Hospital, No. 201, Sec. 2, Shi-Pai Road, Bei-Tou District, Taipei City, Taiwan 11217; 2 Faculty of Medicine, National Yang-Ming University School of Medicine, Taipei, Taiwan; 3 Brain Research Center, National Yang-Ming University, Taipei, Taiwan; 4 Department of Medicine, Taipei City Hospital, Heping Fuyou Branch, Taipei, Taiwan; 5 Research Center of Big Data, College of Management, Taipei Medical University, Taipei, Taiwan

**Keywords:** Diabetic neuropathy, Epilepsy, Fibromyalgia, Neuropathic pain, Persistence, Post-herpetic neuralgia, Pregabalin

## Abstract

**Background:**

Pregabalin is approved for the treatment of neuropathic pain, fibromyalgia, and seizure disorders, although the pivotal trials were mostly carried out in Europe or North America. The prescribing patterns among different indications in Asia have rarely been explored.

**Methods:**

This was a population-based retrospective cohort study based on the National Health Insurance Research Database in Taiwan. Prescriptions of pregabalin were identified, and data regarding demographics, indications, co-existing diagnoses, and concomitant medications were extracted. Pregabalin users were followed for at least one year, and factors associated with persistence at one year were determined by using multivariate logistic regression analysis.

**Results:**

Between June 2012 and December 2016, 114,437 pregabalin users (mean age 60.7 ± 15.4 years, 57.8% female) were identified. The indications included post-herpetic neuralgia (PHN) (30.5%), musculoskeletal diseases other than fibromyalgia (21.2%), fibromyalgia (18.4%), diabetic peripheral neuropathic pain (DPNP) (11.7%) and epilepsy (2.9%). Overall, 62.5% and 6.4% of patients achieved a maximum dose of ≥150 and ≥ 300 mg/day, respectively. The median duration of persistent pregabalin use was 28 days (interquartile range 14–118 days). The one-year persistence rate was 12.1%, and the indications associated with the highest and lowest persistence rates were epilepsy (42.4%) and PHN (6.1%), respectively. Male gender (odds ratio [OR] 1.14, 95% confidence interval [CI] 1.09–1.18), older age (OR 1.01 per year, 95% CI 1.01–1.01), indications other than PHN, especially epilepsy (OR 8.04, 95% CI 7.33–8.81, PHN as reference), and a higher initial dose (OR 1.12 per 75 mg, 95% CI = 1.10–1.15) were associated with persistence at one year, whereas the initial concomitant use of antiviral agents decreased the likelihood (OR 0.41, 95% CI 0.35–0.47).

**Conclusions:**

Pregabalin prescriptions for pain disorders were limited to short-term use, which is consistent around the world. However, the average prescribed dose in Taiwan was lower than those in Western countries, and was frequently below the recommended ranges. Potential causes included the duration of natural history of PHN, and off-label prescriptions for pain in acute herpes zoster, rather than PHN, as well as intolerance to the side effects.

## Introduction

Pregabalin is a new-generation antiepileptic drug targeting at the calcium channel α2-δ subunit, and was approved for the treatment in a number of pain and seizure disorders [1]. In fact, it is recommended as the fist-line agent in the treatment of neuropathic pain (NeP), particularly post-herpetic neuralgia (PHN) and diabetic peripheral neuropathic pain (DPNP) [2–6]. Besides, with regard to the pharmacological treatment in fibromyalgia, pregabalin is one of the drugs of choice according to the evidence available [7–9]. In addition, pregabalin had level A evidence for its efficacy as add-on therapy against treatment-resistant adult focal epilepsy according to the guidelines of the American Academy of Neurology [10]. It was launched in Taiwan in 2009, and became reimbursed for PHN/epilepsy, fibromyalgia, and DPNP by the Taiwan National Health Insurance (NHI) in February 2012, February 2013, and January 2016, respectively. Currently, preparations are available at two dosage forms: 75 mg and 150 mg. The recommended dose ranges are 150-600 mg/day for PHN and epilepsy, 150-450 mg/day for fibromyalgia, and 150-300 mg/day for DPNP [11]. However, the pattern of clinical practice regarding its use in Taiwan had not been explored.

Pregabalin is generally considered well tolerated, and the commonly reported side effects include dizziness, somnolence, peripheral edema, and body weight gain [12]. Although adverse events (AEs) were not uncommon in clinical trials, serious treatment-related AEs were rare. The discontinuation rates due to AEs in the treatment group were either comparable to or only slightly higher than those in the placebo group [10, 13–15]. However, most clinical trials were of relatively short durations and involved a highly selected population, whether the results could reflect what happens in the real-world practice is not without doubt. More importantly, the vast majority of the pivotal trials were carried out in Europe or North America, in which patients of Asian descent were under-presented [12]. Therefore, the present study aimed at evaluating the long-term treatment persistence of pregabalin in the general population by using a nation-wide claims-based dataset in Taiwan.

## Methods

### Data source

This population-based retrospective cohort study was conducted utilizing the NHI Research Database (NHIRD) in Taiwan. The NHI is a social health insurance program integrating all public insurance systems into a single-payer system launched in 1995, and covers more than 99.5% of the entire 23.5 million population for comprehensive health care in Taiwan. NHIRD contains the total registration files and claims data of the NHI, and is maintained by the Taiwan Health and Welfare Data Science Center. These databases provide comprehensive utilization and enrollment information including demographic data, outpatient visit records, hospitalization records, and drug prescription registry for all patients. Diseases were coded according to the 2001 International Classification of Diseases, the Ninth Revision, Clinical Modification (ICD-9-CM) before 2016, and the Tenth Revision (ICD-10) after 2016. The diagnosis codes for the diseases of interest were listed in Supplementary Table.

### Study sample

In this nation-wide population-based retrospective cohort study, all pregabalin prescriptions between February 2012 and December 2016 were identified. The index date was defined as the date of the first pregabalin prescription. Data regarding demographics (including age, sex, monthly income, and residence), diagnoses, and drug prescriptions were extracted, based on which Charlson Comorbidity Index (CCI) scores were calculated. Concomitant use of other medications that could be a confounding factor in the analysis of pregabalin use was also taken into account. Patients with missing data or with follow-up periods less than 12 months were excluded. All subjects were followed until death or the end of the study (Dec 31, 2017).

### Pregabalin prescriptions

For each pregabalin prescription, we collected the information on the drug type, quantity, dispensing date, and days of drug supply. The period of pregabalin use was defined as the duration between the first day of drug prescription and the end of the last-dispensed drug supply. When a patient filled a subsequent prescription within 90 days after the end of the previous supply, he or she was considered to have received continuous therapy. An interval of > 90 days between prescription refills was defined as discontinuation of medication.

### Statistical analysis

Descriptive statistics (means, standard deviations [SDs], and frequencies) were used to characterize the study population at baseline. The Charlson Comorbidity Index score was used to determine the health condition of individual patients. During the follow-up period, we calculated the overall numbers and percentages of persistent pregabalin use. Univariate and multivariate, stepwise, logistic regressions were used to identify the factors in association with continued use of pregabalin at one-year follow-up period. Factors with a *p*-value < 0.1 in the univariate analyses were entered into the multivariate analyses, and the odds ratio (OR) (95% confidence interval, CI) were determined. All statistical analyses were conducted with the SAS 9.4 (SAS Institute. Inc., Cary, NC). A *p*-value < 0.05 was considered to be statistically significant.

## Results

### Characteristics of the study population (Table 1)

During the study period, 114,437 pregabalin users (mean age was 60.7 ± 15.4 years, 57.8% female) were identified. It was mostly commonly prescribed by neurologists (47.1%). The major indications were pain control for PHN (30.5%), musculoskeletal diseases other than fibromyalgia (21.2%), fibromyalgia (18.4%), DPNP (11.7%), and epilepsy (2.9%). The mean initial daily dose of pregabalin was 116.8 ± 58.2 mg per day, and 94.4% were between 75 mg and 225 mg per day. Commonly used concomitant medications included non-steroidal anti-inflammatory drugs (60.0%), acetaminophen (40.1%), acetaminophen/tramadol (27.8%), and others. Besides, anti-viral agents were given along with the initial prescription of pregabalin in 5.8%.

**Table 1:** Baseline characteristics of patients using pregabalin

	Number (percentage)
**No. of patients**	114,437
**Male**	48,316 (42.2)
**Age, mean (SD), year**	60.66 (15.4)
**Monthly income NT$**	
**NT 0–21,009**	36,963 (32.3)
**NT 210,10-36,825**	52,546 (45.9)
**> 36,825**	24,928 (21.8)
**Region of Taiwan**	
**Northern region**	49,791 (43.5)
**Central region**	33,899 (29.6)
**Southern region**	27,122 (23.7)
**Eastern region**	3625 (3.2)
**Charlson Comorbidity Index score**	
**0**	39,922 (34.9)
**1**	26,902 (23.5)
**2–4**	34,764 (30.4)
**> 4**	12,849 (11.2)
**Specialties**	
**Neurology**	53,910 (47.1)
**Anesthesiology**	5541 (4.8)
**Physiatry**	3151 (2.8)
**Psychiatry**	430 (0.4)
**Rheumatology**	9201 (8.0)
**Others**	42,204 (36.9)
**Indications**	
**Herpes zoster**	34,919 (30.5)
**Diabetic peripheral neuropathic pain**	13,369 (11.7)
**Fibromyalgia**	21,051 (18.4)
**Epilepsy**	3301 (2.9)
**Other musculoskeletal diseases**	24,227 (21.2)
**Others**	17,570 (15.4)
**Concomitant medications**	
**Gabapentin**	14,114 (12.3)
**Duloxetine**	4410 (3.9)
**NSAIDs**	68,679 (60)
**Acetaminophen**	45,834 (40.1)
**Tramadol**	5011 (4.4)
**Acetaminophen/tramadol**	31,777 (27.8)
**Lidocaine medicated plaster**	1114 (1.0)
**Anti-viral agents**^a^	6625 (5.8)
**Daily dosage**	
**< 75 mg**	1162 (1.0)
**75-149 mg**	57,106 (49.9)
**150-224 mg**	50,925 (44.5)
**> =225 mg**	5244 (4.6)

^a^Including valaciclovir, ganciclovir, famciclovir, and acyclovir

### Pregabalin prescriptions during the follow-up period

The mean duration of persistent pregabalin use was 149.5 ± 299.8 days (median 28, interquartile range [IQR] 14–118) (Table 2). By 90 days after the index date, 33,570 users (29.3%) were still using pregabalin, and pregabalin use persisted for more than 1 year in 13,885 (12.1%). Among the indications for pregabalin use, epilepsy (42.4%) and DPNP (21.7%) had relatively higher persistence rates by 1 year, with the mean durations of 515.6 ± 603.5 and 222.0 ± 330.1 days, respectively. On the other hand, the one-year persistence rates were lower for fibromyalgia (11.7%) and PHN (6.1%), and the mean durations were 150.8 ± 302.8 and 89.4 ± 224.1 days, respectively. During follow-up, the mean prescribed dose was 140.7 ± 92.8 mg, with the highest and lowest doses for each prescription being epilepsy (200.9 ± 145.4 mg/day) and DPNP (131.2 ± 93.0 mg/day), respectively (Table 3). The maximum dose prescribed was < 150 mg/day in 37.5% of patients, and only 6.4% reached a dose of ≥300 mg/day. However, the maximum dose exceeded 300 mg/day in 24.9% of the prescriptions for epilepsy.

**Table 2:** Persistence of pregabalin during follow-up

		Duration of persistence (days)		No. of patients with persistent pregabalin use			
	No. of patients	Mean (SD)	Median (IQR)	> = 90 days ~ < 180 days	> = 180 days ~ < 1 year	> = 1 year ~ < 2 years	> = 2 years
**All cohort**	114,437	149.5 (299.8)	28 (14–118)	33,570 (29.3)	22,201 (19.4)	13,885 (12.1)	33,570 (29.3)
**Specialties**							
**Neurology**	53,910	179.3 (330.4)	41 (14–161)	18,423 (34.2)	12,623 (23.4)	8165 (15.1)	18,423 (34.2)
**Anesthesiology**	5541	189.8 (358)	42 (14–166)	1928 (34.8)	1302 (23.5)	829 (15)	1928 (34.8)
**Physiatry**	3151	143.4 (301.1)	28 (14–99)	845 (26.8)	566 (18)	364 (11.6)	845 (26.8)
**Psychiatry**	430	222.8 (383.9)	56 (21–209)	162 (37.7)	117 (27.2)	80 (18.6)	162 (37.7)
**Rheumatology**	9201	206.6 (366.3)	47 (21–191)	3444 (37.4)	2388 (26)	1558 (16.9)	3444 (37.4)
**Others**	42,204	93.3 (211.3)	22 (7–69)	8768 (20.8)	5205 (12.3)	2889 (6.8)	8768 (20.8)
**Indications**							
**Herpes zoster**	34,919	89.4 (224.1)	21 (7–56)	6405 (18.3)	3700 (10.6)	2134 (6.1)	6405 (18.3)
**Diabetic peripheral neuropathic pain**	13,369	221.9 (330.1)	71 (28–294)	6044 (45.2)	4381 (32.8)	2894 (21.6)	6044 (45.2)
**Fibromyalgia**	21,051	150.8 (302.8)	28 (14–117)	6179 (29.4)	4026 (19.1)	2470 (11.7)	6179 (29.4)
**Epilepsy**	3301	515.5 (603.5)	220 (35–814)	2085 (63.2)	1748 (53)	1400 (42.4)	2085 (63.2)
**Other musculoskeletal diseases**	24,227	138.3 (269.8)	30 (14–113)	7141 (29.5)	4499 (18.6)	2638 (10.9)	7141 (29.5)
**Others**	17,570	158.6 (295.9)	35 (14–141)	5716 (32.5)	3847 (21.9)	2349 (13.4)	5716 (32.5)

**Table 3:** Proportion of prescribed maximum daily dose of pregabalin during follow-up

		Maximum dose prescribed					
Indications	Mean (SD)	< 150 mg	> = 150 mg ~ < 300 mg	> = 300 mg ~ < 450 mg	> = 450 mg ~ < 600 mg	> = 600 mg	Sum
**Herpes zoster**	147.2 (100.3)	11,257 (32.2)	21,181 (60.7)	1986 (5.7)	359 (1)	136 (0.4)	34,919
**Diabetic peripheral neuropathic pain**	131.2 (93)	5900 (44.1)	6789 (50.8)	580 (4.3)	76 (0.6)	24 (0.2)	13,369
**Fibromyalgia**	139.2 (89)	8323 (39.5)	11,332 (53.8)	1039 (4.9)	294 (1.4)	63 (0.3)	21,051
**Epilepsy**	200.9 (145.4)	763 (23.1)	1717 (52)	504 (15.3)	247 (7.5)	70 (2.1)	3301
**Other musculoskeletal diseases**	131.1 (72.2)	9825 (40.6)	13,483 (55.7)	749 (3.1)	134 (0.6)	36 (0.1)	24,227
**Others**	138.8 (89.1)	6799 (38.7)	9708 (55.3)	823 (4.7)	184 (1)	56 (0.3)	6433
**Total**	140.7 (92.8)	42,867 (37.5)	64,210 (56.1)	5681 (5.0)	1294 (1.1)	385 (0.3)	114,437

### Factors associated with persistent pregabalin use by one year

The persistence rate at 1 year after the index date was 12.1%. In multivariate logistic regression analysis, male gender (OR = 1.14, 95% CI 1.01–1.18, *p* < 0.001), older age (OR = 1.01 per year, 95% CI = 1.01–1.01, *p* < 0.001), higher CCI score, and the initial concomitant prescription with gabapentin, duloxetine, tramadol, acetaminophen/tramadol, and lidocaine medicated plaster were associated with a significantly increased probability of persistent use of pregabalin at 1 year (Table 4). Compared with prescriptions from neurologists, those from rheumatologists (OR = 1.64, 95% CI = 1.54–1.76, *p* < 0.001) and psychiatrists (OR = 1.31, 95% CI = 1.01–1.70, *p* = 0.039) were more likely to be persistent at 1 year. Among the different indications of pregabalin prescription, epilepsy (OR = 8.04, 95% CI = 7.33–8.81, *P* < 0.001) was associated with the highest probability of continued use, followed by DPNP (OR = 3.05, 95% CI = 2.85–3.27, *p* < 0.001), other musculoskeletal diseases (OR = 1.64, 95% CI = 1.54–1.75, *p* < 0.001), and fibromyalgia (OR = 1.59, 95% CI = 1.48–1.70, *p* < 0.001), when compared to PHN. Besides, a higher initial pregabalin dose (OR = 1.12 per 75 mg, 95% CI = 1.10–1.15, *p* < 0.001) was also associated with an increased probability of persistence at 1 year. On the other hand, concomitant prescription of anti-viral agents was associated with decreased likelihood of pregabalin use at 1 year (OR = 0.41, 95% CI = 0.35–0.47, *p* < 0.001).

**Table 4:** Factor associated with persistent use of pregabalin at one year

	Univariate		Multivariable	
Variable	Odds Ratio (95% CI)	*p* Value	Odds Ratio (95% CI)	*p* Value
**Male**	1.24 (1.19–1.28)	<.001	1.14 (1.09–1.18)	< 0.001
**Age, per year**	1 (1–1)	0.001	1.01 (1.01–1.01)	< 0.001
**Monthly income NT$**				
**NT 0–21,009**	1		1	
**NT 210,10-36,825**	0.87 (0.84–0.91)	< 0.001	0.97 (0.93–1.01)	0.187
**> 36,825**	0.75 (0.71–0.79)	< 0.001	0.87 (0.83–0.92)	< 0.001
**Region of Taiwan**				
**Northern region**	1		1	
**Central region**	0.94 (0.9–0.98)	0.007	0.86 (0.82–0.9)	< 0.001
**Southern region**	0.92 (0.83–1.02)	0.126	0.79 (0.71–0.89)	< 0.001
**Eastern region**	0.9 (0.86–0.94)	< 0.001	1 (0.95–1.05)	0.981
**Charlson Comorbidity Index score, per 1 score**	1.06 (1.05–1.06)	< 0.001		
**Specialty of prescribing physicians**				
**Neurology**	1		1	
**Anesthesiology**	0.99 (0.91–1.07)	0.715	1.08 (0.99–1.17)	0.094
**Physiatry**	0.73 (0.65–0.82)	< 0.001	0.92 (0.82–1.03)	0.141
**Psychiatry**	1.28 (1–1.64)	0.047	1.31 (1.01–1.7)	0.039
**Rheumatology**	1.14 (1.08–1.21)	< 0.001	1.64 (1.54–1.76)	< 0.001
**Others**	0.41 (0.39–0.43)	< 0.001	0.61 (0.58–0.64)	< 0.001
**Indications**				
**Herpes zoster**	1		1	
**Diabetic peripheral neuropathic pain**	4.24 (4–4.51)	< 0.001	3.05 (2.85–3.27)	< 0.001
**Fibromyalgia**	2.04 (1.92–2.17)	< 0.001	1.59 (1.48–1.7)	< 0.001
**Epilepsy**	11.31 (10.43–12.28)	< 0.001	8.04 (7.33–8.81)	< 0.001
**Other musculoskeletal diseases**	1.88 (1.77–1.99)	< 0.001	1.64 (1.54–1.75)	< 0.001
**Others**	2.37 (2.23–2.52)	< 0.001	1.98 (1.85–2.11)	< 0.001
**Concomitant medications**				
**Gabapentin**	2.04 (1.95–2.14)	< 0.001	2.04 (1.94–2.15)	< 0.001
**Duloxetine**	2.17 (2.02–2.33)	< 0.001	1.53 (1.42–1.65)	< 0.001
**NSAIDs**	0.56 (0.54–0.58)	< 0.001	0.72 (0.7–0.75)	< 0.001
**Acetaminophen**	0.61 (0.59–0.64)	< 0.001	0.79 (0.76–0.83)	< 0.001
**Tramadol**	1.43 (1.32–1.54)	< 0.001	1.3 (1.2–1.42)	< 0.001
**Acetaminophen/tramadol**	0.99 (0.95–1.03)	0.620	1.08 (1.03–1.12)	0.001
**Lidocaine medicated plaster**	1.2 (1.01–1.42)	0.035	1.37 (1.15–1.64)	0.001
**Anti-viral agents**	0.22 (0.19–0.25)	< 0.001	0.41 (0.35–0.47)	< 0.001
**Daily dosage, per 75 mg**	1.16 (1.13–1.19)	< 0.001	1.12 (1.10–1.15)	< 0.001

## Discussion

Pregabalin was most commonly prescribed for the treatment of pain in PHN (30.5%) and fibromyalgia (18.4%) in Taiwan, and this was also reflected by a high proportion of concomitant prescription of other analgesics (Table 1). Most of the prescriptions were limited to short-term use, as evidenced by a mean duration of prescription lasting 149.5 ± 299.8 days (median 28, interquartile range [IQR] 14–118), especially in PHN (89.4 ± 224.1 days) and fibromyalgia (150.8 ± 302.8 days). Besides, the mean dose prescribed was 140.7 ± 92.8 mg, which was lower than the doses used in clinical trials for NeP [3] and fibromyalgia [8], although the mean dose of prescriptions for epilepsy could be within the lower range of recommended doses [10].

The finding that pregabalin prescriptions were typically of short duration is in keeping with prior reports. According to a retrospective administrative claims data analysis from the United States, only 56.9% of patients initiating pregabalin for PHN obtained a refill during the one-year follow-up period [16]. Similarly, 34% had just one single dispensed prescription for various indications, including epilepsy, generalized anxiety disorder, and neuropathic pain, indicating only 64% had further refills after the first prescription in Sweden [17]. Besides, according to an Israeli study in fibromyalgia, the median time to discontinuation for antiepileptics, namely pregabalin and gabapentin, was 30 days (IQR 30-106 days) [18], and the mean prescription duration of pregabalin for pain was 52.2 days in a Japanese retrospective real-world study [19]. In addition, similar findings were reported in prospective real-world studies involving patients with NeP. The median durations of pregabalin use were 84.0 days in a Latin American multicenter, open-label study [20], and 62 days in a Greek non-interventional, multicenter study [21]. In the present study, the persistence rates were 29.3% and 12.1% at 90 days and one year, respectively, indicating only a minority of patients continued to use pregabalin. All the above findings indicate that pregabalin prescriptions are usually discontinued early, which is a phenomenon commonly seen in different parts of the world.

A number of factors could be responsible for the short duration of pregabalin treatment for pain. As the NeP in PHN could resolve in months or years [22, 23], treatment would be no longer necessary after resolution of symptoms. In fact, the prevalence of PHN was 11.6%, 8.5%, 7.4%, and 6.0% at months 3, 6, 9, and 12 following herpes zoster [22], and the natural course of PHN could be one of the major causes for the short-term use of pregabalin in that patient population. More importantly, pregabalin could be prescribed off-label for the acute pain in herpes zoster rather than for PHN in some patients in the present cohort. In fact, 5.8% of pregabalin users were prescribed with anti-viral agents at the same time (Table 1), and concomitant use of anti-viral agents was associated with decreased likelihood of pregabalin use at 1 year (OR 0.41, 95% CI 0.35–0.47, *p* < 0.001) (Table 4). Although pregabalin is only indicated for PHN, there is some evidence supporting its efficacy for acute pain of herpes zoster [24, 25]. On the other hand, pregabalin could also be discontinued due to a lack of desired efficacy or the development of intolerable side effects. In fact, adverse effects of pregabalin treatment usually appear early in the treatment course, and usually resolve after prolonged use [11]. Premature discontinuation preclude further escalation of the dosage, and thus could also account for the finding that most prescriptions were under-dosed, especially in pain disorders. Both could lead to poor treatment outcomes and hence compromised quality of life. An in-depth understanding of the dynamics of development of adverse events, would be helpful to optimize the prescription of pregabalin.

Among the different indications included, epilepsy patients had the greatest likelihood of long-term use, and PHN the least. Similar findings were reported in the Swedish study mentioned above [17], in which epilepsy and NeP had the best and worst persistence rates during follow-up. Generally speaking, studies evaluating drug treatment in epilepsy usually report high persistence or adherence rates [26–28]. This could probably be attributed to the unremitting nature of most seizure disorders, as well as the devastating and potentially life-threating consequences of not receiving treatment. In contrast, a substantial proportion of PHN resolves within months [22, 23], and consequently, treatment for pain would be no longer necessary at that time. Taken together, the durations of the underlying diseases could have an important impact on the durations of pregabalin prescriptions. Besides, as in the American study mentioned above, the low persistence rate of pregabalin use in the treatment of NeP could also be attributed to suboptimal dosing, with 87 % reaching ≥ 150 mg/day and 27 % reaching ≥ 300 mg/day [16], which were higher than the corresponding figures of 62.5% and 6.4%, respectively, in the present study (Table 3). In fact, most of the prescriptions conformed well to the treatment recommendations. However, more than a third of the prescriptions for pain control were <150 mg/day, which was below the lowest recommended dose, particularly in prescriptions for DPNP (44.1%) and fibromyalgia (39.5%) (Table 3). Besides, the average doses prescribed for NeP (147.2±100.3 mg for PHN, 131.2±93 mg for DPNP) (Table 3) were lower than those in the American (187 mg for PHN) [16] and Swedish studies (275 mg for NeP) [17]. In comparison, the findings were variable in prospective open-label studies. Although the doses prescribed fell in the lower range of the recommended doses in some studies, the doses were still higher than those in the current study, possibly with the exception of the Greek study, in which the most commonly prescribed dose was 150 mg/day [21]. On the other hand, the mean daily dose was 301 mg in a German open-label multicenter study for patients with DPNP and PHN [29]. Besides, the average doses in the first, second, and third month and after three months of treatment for NeP were 148.4, 145.3, 169.3, and 240.7 mg/day, respective, in a Danish study of peripheral NeP [30], and 42.6% and 38.9% of patients achieved a daily dose of 300 mg and 600 mg, respectively, in the Latin American NeP study [20]. The exact cause why the average dose used in the current study was lower than in some earlier reports was unknown. It could be possible that some patients could benefit from such low doses, which made further titration unnecessary. This might be the case for DPNP, as pregabalin prescriptions in such patients were characterized by a long duration (221.9±330.1 days) (Table 2) despite a relatively lower dose (131.2±93 mg) (Table 3). However, it could also be possible that higher doses of pregabalin were not that well tolerated in some patients. In such cases, there could be inadequate therapeutic effects at lower doses, although dose escalation might be precluded by side effects. In addition, ethnic factors could also play a role. The tolerability of pregabalin was reported to be comparable to, or slightly worse than, that of placebo in clinical trials [10, 13–15], which were mostly carried out in North America and/or Europe. In fact, side effects, such as dizziness, somnolence, peripheral edema, and weight gain, were more common among Asians as compared to Caucasians [12]. This could result from increased exposure to pregabalin due to the relatively lower average body weights among Asian patients, which could result in decreased tolerability, and possibly lower average doses prescribed. On the other hand, although ethnicity did not seem to have a major impact on the pharmacokinetics of pregabalin [31], it remains possible there could be ethnic differences in the pharmacodynamics.

The most important strength of the present study is the large-scale sample recruited, and the almost complete coverage of our NHI leads to the relative freedom from selection bias of the dataset. Besides, the data from NHIRD have been validated by a number of studies, and have yielded important findings that are widely accepted [32–34]. On the other hand, the main weaknesses include the validity of the diagnosis coding and the lack of relevant clinical information regarding the initiation and discontinuation of the prescription. PHN, DPNP, and fibromyalgia could not be accurately coded in the ICD-9-CM coding system, and this remains an issue in the ICD-10 coding system, except for fibromyalgia. Besides, it could be possible that fibromyalgia could be miscoded as other musculoskeletal diseases. In addition, as discussed, some of the patients categorized as PHN could actually be treated for acute pain in herpes zoster. However, when these diagnosis codes were associated with pregabalin prescriptions concomitantly, the probability that these codes correspond to the designated diagnoses should be high. In addition, the exact cause for treatment discontinuation could not be ascertained as such information is not available in the NHIRD. In fact, this is an intrinsic weakness for studies based on administrative claims databases of health insurance or drug prescription register [16–18], and it is unknown whether the discontinuation of prescriptions of pregabalin was attributed to either efficacy or tolerability issues. Moreover, based on the methodology of the current study, medications prescribed on the same day would be categorized as concomitant, even though the prescriptions might not be necessarily given at the same consultation or even from the same physician. Although the medications could be adjusted or changed within the same day, the underlying diagnoses would remain the same. Therefore, such a methodological issue would probably have little impact on the analysis. Despite the above weaknesses, the present study could still provide some useful information regarding the pattern of pregabalin prescriptions in our country.

## Conclusion

In conclusion, pain control in PHN and fibromyalgia was the most common indication for pregabalin use in Taiwan, although many of the prescriptions were limited to low dosage and short-term use. In addition to the duration of natural history of PHN, other potential causes included off-label prescription for acute pain in herpes zoster, as well as an increased incidence of side effects in Asian populations. A thorough understanding of the temporal profile of adverse effects, as well as education for both the clinicians and patients, could help circumvent premature discontinuation and could therefore optimize the treatment outcome.

## Supplementary information

**Additional file 1: Supplementary Table.** The ICD diagnosis codes of indications for pregabalin

## Data Availability

The NHIRD is publicly available from the Taiwan NHI.

## Abbreviations

CCI: Charlson comorbidity index
CI: Confidence interval
DPNP: Diabetic peripheral neuropathic pain
ICD-9-CM: International classification of diseases, the ninth revision, clinical modification
IQR: Interquartile range
OR: Odds ratio
NeP: Neuropahic pain
NHI: National health insurance
NHIRD: National health insurance research database
PHN: Post-herpetic neuralgia
SD: Standard deviation

## References

[1] Calandre EP, Rico-Villademoros F, Slim M (2016). Alpha2delta ligands, gabapentin, pregabalin and mirogabalin: a review of their clinical pharmacology and therapeutic use. Expert Rev Neurother.

[2] Dworkin RH, O'Connor AB, Backonja M, Farrar JT, Finnerup NB, Jensen TS (2007). Pharmacologic management of neuropathic pain: evidence-based recommendations. Pain.

[3] Finnerup NB, Attal N, Haroutounian S, McNicol E, Baron R, Dworkin RH (2015). Pharmacotherapy for neuropathic pain in adults: a systematic review and meta-analysis. Lancet Neurol.

[4] Dworkin RH, O'Connor AB, Audette J, Baron R, Gourlay GK, Haanpaa ML (2010). Recommendations for the pharmacological management of neuropathic pain: an overview and literature update. Mayo Clin Proc.

[5] Bril V, England J, Franklin GM, Backonja M, Cohen J, Del Toro D (2011). Evidence-based guideline: treatment of painful diabetic neuropathy: report of the American Academy of Neurology, the American Association of Neuromuscular and Electrodiagnostic Medicine, and the American Academy of physical medicine and rehabilitation. Neurology..

[6] Bril V, England JD, Franklin GM, Backonja M, Cohen JA, Del Toro DR (2011). Evidence-based guideline: treatment of painful diabetic neuropathy--report of the American Association of Neuromuscular and Electrodiagnostic Medicine, the American Academy of Neurology, and the American Academy of Physical Medicine & Rehabilitation. Muscle Nerve.

[7] Calandre EP, Rico-Villademoros F, Slim M (2015). An update on pharmacotherapy for the treatment of fibromyalgia. Expert Opin Pharmacother.

[8] Macfarlane GJ, Kronisch C, Dean LE, Atzeni F, Hauser W, Fluss E (2017). EULAR revised recommendations for the management of fibromyalgia. Ann Rheum Dis.

[9] Arnold LM, Clauw DJ (2017). Challenges of implementing fibromyalgia treatment guidelines in current clinical practice. Postgrad Med.

[10] Kanner AM, Ashman E, Gloss D, Harden C, Bourgeois B, Bautista JF (2018). Practice guideline update summary: efficacy and tolerability of the new antiepileptic drugs II: treatment-resistant epilepsy: report of the guideline development, dissemination, and implementation Subcommittee of the American Academy of neurology and the American Epilepsy Society. Neurology..

[11] Pregabalin package insert (2019). https://info.fda.gov.tw/MLMS/ShowFile.aspx?LicId=02024995&Seq=015&Type=9.

[12] Ogawa S, Satoh J, Arakawa A, Yoshiyama T, Suzuki M (2012). Pregabalin treatment for peripheral neuropathic pain: a review of safety data from randomized controlled trials conducted in Japan and in the west. Drug Saf.

[13] Waldfogel JM, Nesbit SA, Dy SM, Sharma R, Zhang A, Wilson LM (2017). Pharmacotherapy for diabetic peripheral neuropathy pain and quality of life: a systematic review. Neurology..

[14] Straube S, Derry S, Moore RA, McQuay HJ (2010). Pregabalin in fibromyalgia: meta-analysis of efficacy and safety from company clinical trial reports. Rheumatology (Oxford).

[15] Hauser W, Bernardy K, Uceyler N, Sommer C (2009). Treatment of fibromyalgia syndrome with gabapentin and pregabalin--a meta-analysis of randomized controlled trials. Pain.

[16] Johnson P, Becker L, Halpern R, Sweeney M (2013). Real-world treatment of post-herpetic neuralgia with gabapentin or pregabalin. Clin Drug Investig.

[17] Wettermark B, Brandt L, Kieler H, Boden R (2014). Pregabalin is increasingly prescribed for neuropathic pain, generalised anxiety disorder and epilepsy but many patients discontinue treatment. Int J Clin Pract.

[18] Ben-Ami Shor D, Weitzman D, Dahan S, Gendelman O, Bar-On Y, Amital D (2017). Adherence and persistence with drug therapy among fibromyalgia patients: data from a large health maintenance organization. J Rheumatol.

[19] Ushida T, Matsui D, Inoue T, Yokoyama M, Takatsuna H, Matsumoto T (2019). Recent prescription status of oral analgesics in Japan in real-world clinical settings: retrospective study using a large-scale prescription database. Expert Opin Pharmacother.

[20] Xochilcal-Morales M, Castro EM, Guajardo-Rosas J, Obregon TN, Acevedo JC, Chucan JM (2010). A prospective, open-label, multicentre study of pregabalin in the treatment of neuropathic pain in Latin America. Int J Clin Pract.

[21] Anastassiou E, Iatrou CA, Vlaikidis N, Vafiadou M, Stamatiou G, Plesia E (2011). Impact of pregabalin treatment on pain, pain-related sleep interference and general well-being in patients with neuropathic pain: a non-interventional, multicentre, post-marketing study. Clin Drug Investig..

[22] Bouhassira D, Chassany O, Gaillat J, Hanslik T, Launay O, Mann C (2012). Patient perspective on herpes zoster and its complications: an observational prospective study in patients aged over 50 years in general practice. Pain.

[23] McKendrick MW, Ogan P, Care CC (2009). A 9 year follow up of post herpetic neuralgia and predisposing factors in elderly patients following herpes zoster. J Inf Secur.

[24] Jensen-Dahm C, Rowbotham MC, Reda H, Petersen KL (2011). Effect of a single dose of pregabalin on herpes zoster pain. Trials..

[25] Kanodia SK, Singhal KC (2011). A study on efficacy of Pregabalin in acute herpetic neuralgia. Ann Neurosci.

[26] Faught E, Helmers S, Thurman D, Kim H, Kalilani L (2018). Patient characteristics and treatment patterns in patients with newly diagnosed epilepsy: a US database analysis. Epilepsy Behav.

[27] Aylward BS, Rausch JR, Modi AC (2015). An examination of 1-year adherence and persistence rates to antiepileptic medication in children with newly diagnosed epilepsy. J Pediatr Psychol.

[28] Lee YK, Ah YM, Choi YJ, Cho YS, Kim KJ, Lee JY (2016). Antiepileptic drug adherence and persistence in children with epilepsy attending a large tertiary care children's hospital. Epileptic Disord.

[29] Baron R, Brunnmuller U, Brasser M, May M, Binder A (2008). Efficacy and safety of pregabalin in patients with diabetic peripheral neuropathy or postherpetic neuralgia: open-label, non-comparative, flexible-dose study. Eur J Pain.

[30] Crawford ME, Poulsen PB, Schiottz-Christensen B, Habicht A, Strand M, Bach FW (2016). Real-life efficacy of pregabalin for the treatment of peripheral neuropathic pain in daily clinical practice in Denmark: the NEP-TUNE study. J Pain Res.

[31] Shoji S, Suzuki M, Tomono Y, Bockbrader HN, Matsui S (2011). Population pharmacokinetics of pregabalin in healthy subjects and patients with post-herpetic neuralgia or diabetic peripheral neuropathy. Br J Clin Pharmacol.

[32] Shih CJ, Chu H, Chao PW, Lee YJ, Kuo SC, Li SY (2014). Long-term clinical outcome of major adverse cardiac events in survivors of infective endocarditis: a nationwide population-based study. Circulation..

[33] Wang YF, Chen YT, Luo JC, Chen TJ, Wu JC, Wang SJ (2017). Proton-pump inhibitor use and the risk of first-time ischemic stroke in the general population: a Nationwide population-based study. Am J Gastroenterol.

[34] Peng KP, Chen YT, Fuh JL, Tang CH, Wang SJ (2017). Migraine and incidence of ischemic stroke: a nationwide population-based study. Cephalalgia.

