# Supplementary material for: Persistence of pregabalin treatment in Taiwan: a nation-wide population-based study
Source: J Headache Pain. 2020 May 19;21(1):54. doi: 10.1186/s10194-020-01123-4 (PMC7236209; doi:10.1186/s10194-020-01123-4)
Supplement: Supplementary file 1 — Additional file 1: Supplementary Table. The ICD diagnosis codes of indications for pregabalin [file 10194_2020_1123_MOESM1_ESM.docx]

| **Supplementary Table. The ICD diagnosis codes of indications for pregabalin.** | | |
| --- | --- | --- |
|  | **ICD-9-CM codes** | **ICD-10 codes** |
| **Herpes zoster** | **053.x** | **B02.x, B00.9** |
| **Diabetic peripheral neuropathic pain** | **250.x** | **E08.x, E09.x, E10.x, E11.x, E13.x** |
| **Fibromyalgia** | **729.x** | **M60.8x, M60.9, M79.0, M79.1, M79.7, M79.9** |
| **Epilepsy** | **345.x, 780.3, 780.39** | **G40.x, R56.1, R56.9** |
| **Other musculoskeletal diseases** | **710.x-739.x, except 729.x** | **M05.x, M06.x, M12.x, M13.x, M15.x, M17.x, M19.x, M35.x, M43.x, M45.x, M46.x, M47.x, M48.x, M50.x, M79.x (excluding M79.7)** |
|  |  | |
